# Dementia screening in the community using a brief dementia test with subjective and objective measures

**DOI:** 10.1002/alz70860_097378

**Published:** 2025-12-23

**Authors:** Vanessa Lunardi, Jing Wei Lim

**Affiliations:** ^1^ Khoo Teck Puat Hospital, Singapore, Singapore

## Abstract

**Background:**

Commonly used dementia screening tools include objective cognitive measures such as the Mini‐Mental State Examination (MMSE) and subjective reports of functional decline such as the AD8 Dementia Screening Interview (AD8). However, such screening tests are subject to time constraints when conducted in the primary care setting and the community. Our previous study has shown that a brief dementia test (BDT), consisting of AD8 and MMSE subcomponents, namely three‐item recall and intersecting pentagon copy, demonstrates excellent diagnostic utility and consistency with dementia diagnosis. However, the BDT has not been validated in the setting of a health screening. In this pilot trial, we applied the relatively shorter BDT as a dementia screening tool and verified its performance against a full MMSE on community‐dwelling elderly adults.

**Method:**

MMSE was administered to community‐dwelling Singaporean elderly (*n* = 18, age 63 to 80) and AD8 was completed by their accompanying informant or self‐rated when an informant was unavailable. The AD8 score indicates the total number of functional deficits reported (score range 0‐8). The MMSE score indicates the number of correct responses (score range 0‐30). The BDT score is determined by summation of the reversed AD8 scores with the two MMSE subcomponents (score range 0‐12). Optimal cut‐offs for the BDT, MMSE and AD8 were 8/9, 25/26 and 1/2 respectively as determined by our previous study.

**Result:**

The BDT demonstrated strong, positive correlation with the full 30‐item MMSE (Pearson's correlation coefficient = 0.753, *p* <0.001). Percentage of relative standard deviation for the BDT (11.1±13.0%;) was similar to the MMSE (27.1±13.1%).

**Conclusion:**

This first application of the BDT as a dementia screening tool in the community indicates that the BDT may be a robust, time‐saving alternative to the MMSE to assess cognitive function in primary care and community settings with limited resources.

